# Phenotypic Characterization of Metastatic Anaplastic Thyroid Cancer Stem Cells

**DOI:** 10.1371/journal.pone.0065095

**Published:** 2013-05-28

**Authors:** Wen Li, Ashley N. Reeb, William A. Sewell, George Elhomsy, Reigh-Yi Lin

**Affiliations:** 1 Department of Internal Medicine, Division of Endocrinology, Saint Louis University School of Medicine, Saint Louis, Missouri, United States of America; 2 Saint Louis University Cancer Center, Saint Louis, Missouri, United States of America; Consiglio Nazionale delle Ricerche (CNR), Italy

## Abstract

Emerging evidence suggests cancer stem cells (CSCs) may initiate new tumors in anaplastic thyroid carcinoma (ATC), one of the most aggressive solid tumors in humans. However, the involvement of CSCs in human tumorigenesis has not been previously studied in authenticated ATC cell lines. Here we demonstrate a functional role of CSCs in four new validated human ATC cell lines (THJ-11T, THJ-16T, THJ-21T and THJ-29T). We identified and enriched CSCs using a spheroid-forming assay. About 3 to 9% of cells from four ATC cell lines formed thyrospheres. The thyrospheres expressed the stem cell markers NANOG and Oct4 and possessed the ability to self-renew. Injection of these thyrospheres into the thyroids of NOD/SCID *Il2rg-/-* mice resulted in the formation of metastatic tumors that recapitulated the clinical features of human ATC. To our knowledge, this is the first *in vivo* characterization of thyroid CSCs using validated human ATC cell lines. The availability of disease-specific thyrospheres and our orthotopic tumor models will enable the elucidation of disease mechanisms and the environmental niche of CSCs. They may also be useful for preclinical therapeutic screening and for monitoring the effects of biological therapies on ATC.

## Introduction

Anaplastic thyroid carcinoma (ATC) is one of the most lethal human malignancies. Ninety percent of patients with ATC die within six months of diagnosis. Although it is relatively rare — it accounts for only 2% of all thyroid cancer cases — ATC causes more than 50% of all thyroid cancer deaths every year. Current treatments for ATC are aggressive, and include surgery, chemotherapy and radiation therapy. However, ATC is resistant to all types of therapy and disease prognosis has remained unchanged for more than 50 years [1]. Clearly, ATC is a major diagnostic and therapeutic challenge.

A subset of cancer stem cells (CSCs) has been hypothesized to reconstitute and sustain tumor growth in ATC [2]–[7]. CSCs are tumor-initiating cells that possess stem-cell-like properties. They are characterized by an ability to undergo both symmetrical and asymmetrical division, as well as to differentiate into a multitude of tumor cell types. They are largely quiescent, which allows them to escape standard chemotherapies aimed at rapidly dividing cells. CSCs can be isolated from both established thyroid cancer cell lines and tumor specimens. However, a serious cell-line contamination issue has cast doubt on the results of many recent studies of human ATC cell lines, including two in which we and other laboratories described a CD133-positive CSC population in the ATC cell line ARO that was both tumorigenic and resistant to chemotherapy [8], [9]. This ARO cell line has since been shown to be contaminated with the human colon cancer cell line HT-29. In fact, Schweppe *et al* found that up to 42% of the thyroid cancer cell lines commonly used in thyroid research during the past two decades are misidentified, redundant or cross-contaminated [10]. This alarming discovery has driven the thyroid research community to create new, validated thyroid cancer cell lines.

In this study, we evaluated four authenticated human ATC cell lines (THJ-11T, THJ-16T, THJ-21T and THJ-29T) for the existence of CSCs that can initiate neoplastic growth. As detailed in a report by Marlow *et al*, all four human ATC cell lines were established from tumors removed from ATC patients and were examined for the presence of known thyroid tumorigenesis changes, including mutations in *BRAF, KRAS, NRAS*, and *HRAS*; mutations in the *RET/PTC1, RET/PTC2*, and/or *RET/PTC3* fusion-oncogenes; and any of the known variants of the *PAX8/PPARγ* fusion-oncogene [11]. Unique genetic mutations and 12 short tandem DNA repeat (STR) sequences were used to link the cell lines to their respective tumors — making them the first set of thyroid cancer cell lines that can be incontrovertibly traced to their tissue of origin [11].

To examine whether a CSC population exists in these cell lines, we first established a reliable spheroid-forming assay to identify and enrich thyroid CSCs. Next, we tested the effect of the chemotherapy agent cisplatin on these cell lines. We then assessed the ability of these cells to initiate new tumors during serial *in vivo* passaging at limiting dilutions in immunodeficient NOD/SCID *Il2rg-/-* mice. Finally, we explored the metastatic potential of ATC thyrospheres in two xenotransplantation models: orthotopic thyroid transplantation to determine whether the thyrospheres are tumorigenic and capable of invading local tissues, and a tail-vein injection model of experimentally induced lung metastasis to test the ability of the cells to metastasize to distant sites.

## Results

### ATC cells maintain clonogenic capacity *in vitro*

**Figure 1:**
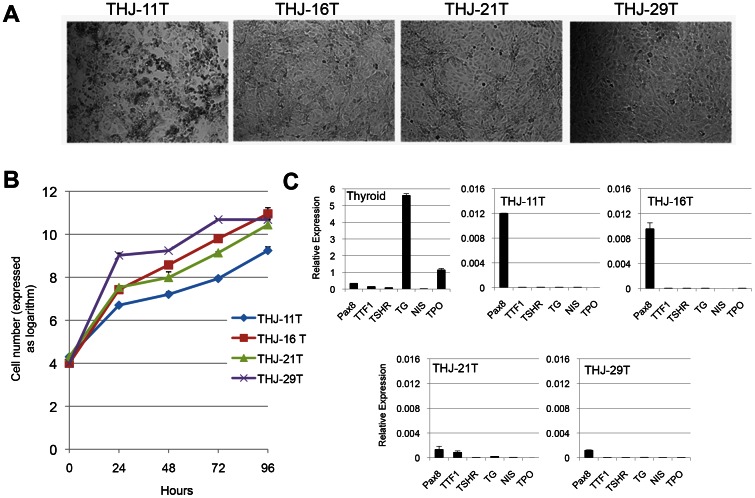
Characterization of ATC cells *in vitro*. (A) Phase contrast microscopy images of four ATC cell lines cultured as monolayers in RPMI medium. (B) Growth proliferation curve over 96 hours demonstrates the proliferative potential of these ATC cell lines. (C) qRT-PCT analysis of thyroid transcription factors *Pax8* and *TTF1* and thyroid differentiation markers *TSHR*, *TG*, *NIS*, and *TPO*. Human GAPDH was used as the housekeeping gene during the amplifications.

We first studied the proliferation of four human ATC cell lines: THJ-11T (p67), THJ-16T (p88), THJ-21T (p56) and THJ-29T (p89). The phase contrast images of these cell lines cultured in RPMI medium as monolayers and the cell proliferation curve over a 96-hour period are shown in Fig. 1A and 1B. Real-time quantitative reverse transcriptase-PCR (qRT-PCR) analysis showed that all cell lines expressed paired box gene 8 (*Pax8*), a thyroid-specific transcription factor. One of the cell lines, THJ-21T, also expressed thyroid transcription factor 1 (*TTF1*). In contrast, none of the cell lines expressed thyroid differentiation markers such as thyroglobulin (*Tg*), sodium/iodide symporter (*NIS*), thyroid peroxidase (*TPO*) or the receptor for thyroid stimulating hormone (*TSHR*) - confirming the dedifferentiated state of these ATC cell lines (Fig. 1C).

**Figure 2:**
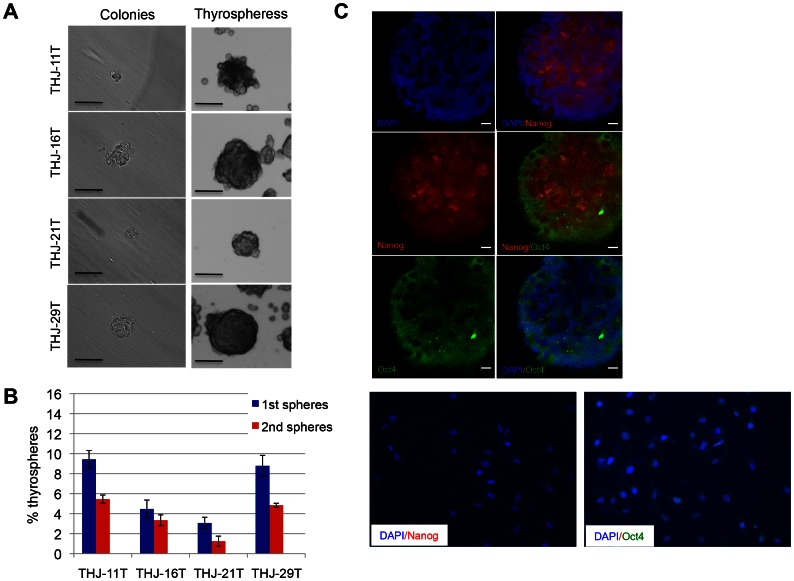
ATC cells retain self-renewal capacity *in vitro*. (A) Representative phase contrast microscopy analysis of colonies (left) and thyrospheres (right) after seven days of culture. Scale bar, 100 µm. (B) Percentage of primary and secondary thyrospheres in all four ATC cell lines. (C) Representative confocal microscopy images of THJ-21T thyrospheres indicated the expression of Nanog (red), Oct4 (green) and DAPI (blue). Scale bar, 10 µm (upper panel). Representative phase contrast images of THJ-21T parental monolayer cells indicated the expression of Nanog and Oct4 is unique to thyrospheres and is not seen in the parental monolayer cells (lower panel).

To characterize CSCs in these cell lines, we assessed their ability to form colonies and thyrospheres *in vitro*. Fig. 2A shows that all cell lines formed colonies in methylcellulose-based media after seven days in culture. They also formed free-floating thyrospheres when seeded in stem-cell-culture conditions on ultra-low attachment plates. Limiting dilution analysis indicated that the mean percentage of thyrospheres formed was 9.4 ± 0.8% in THJ-11T cells, 4.5 ± 0.9% in THJ-16T cells, 3.1 ± 0.6% in THJ-21T cells and 8.8 ± 1.0% in THJ-29T cells (Fig. 2B). The diameter of primary thyrospheres ranged from 100 to 150 µm. These thyrospheres can be expanded after several passages. The mean percentage of secondary thyrospheres was reduced in all cell lines: 5.5 ± 0.4% in THJ-11T cells, 3.3 ± 0.5% in THJ-16T cells, 1.2±0.5% in THJ-21T cells, and 4.9 ± 0.2% in THJ-29T cells (Fig. 2B). This reduction may be the result of an initial phase of symmetric expansion of the seeded thyroid stem cells, followed by the asymmetric division that gives rise to the differentiated progeny that make up the bulk of the thyrosphere cells. We next assessed by indirect immunofluorescence the expression of the pluripotency markers Nanog and Oct4 in thyrospheres generated from all four ATC cell lines. Representative confocal microscopy images of THJ-21T thyrospheres indicated expression of Nanog and Oct4 (Fig. 2C). Representative phase contrast images of THJ-21T parental monolayer cells indicated that the expression of these stem cell markers is unique to thyrospheres, and as noted above, is not seen in the parental monolayer cells. These observations suggest that all ATC cell lines have the ability to self-renew *in vitro*. Furthermore, there is clear evidence of the expression of stem-cell-associated genes in the thyrospheres.

### The effect of the chemotherapeutic agent cisplatin on ATC cells

**Figure 3:**
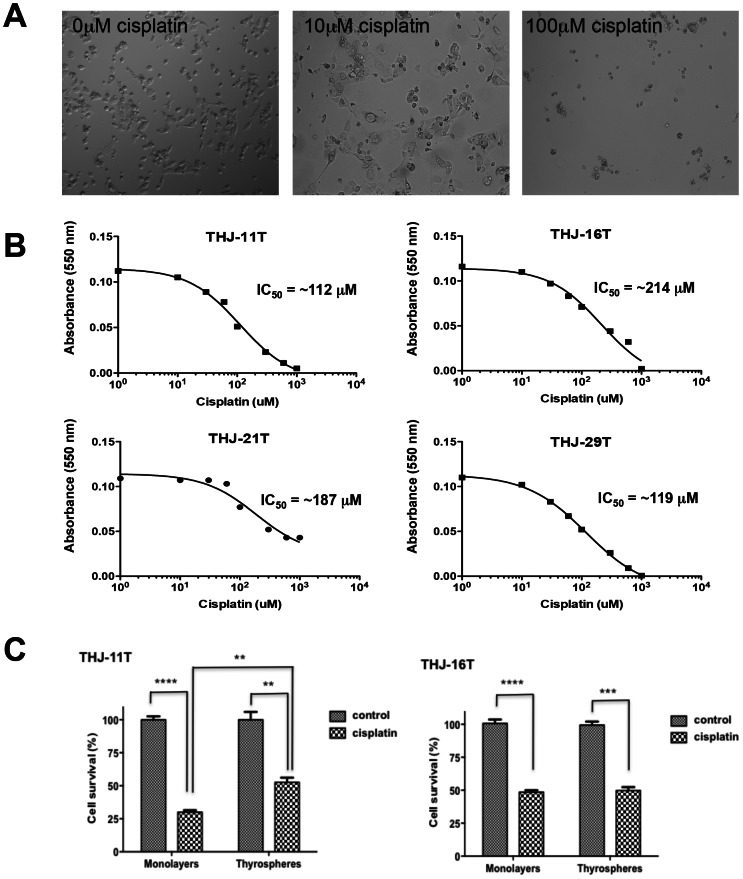
The effect of cisplatin on ATC cells. (A) Representative phase contrast microscopy images of THJ-11T parental monolayer-derived cells exposed to the indicated dose of cisplatin for 48 hours. (B) Dose-dependent inhibition of growth in all ATC cell lines. IC_50_ values are as shown for each cell line. (C) Comparison of *in vitro* resistance to cisplatin of the parental monolayer cells and spheroid-forming cells. The THJ-11T and THJ-16T parental monolayer-derived cells and thyrosphere-derived cells were treated with 10 µM of cisplatin and the fraction of proliferating cells remaining was subsequently estimated based on the Alamar Blue cell proliferation assay. All experiments were performed in triplicate. ****, *P*<0.0001; ***, *P*<0.001; **, *P*<0.05.

We examined the effect of the chemotherapeutic agent cisplatin, which is currently being tested in a Phase I/II clinical trial in patients with ATC, on our ATC cell lines. Fig. 3A shows representative phase contrast microscopy images of THJ-11T cells exposed to the indicated dose of cisplatin for 48 hours. Our results demonstrate a dose-responsive inhibition by cisplatin of cell growth and established an IC_50_ for each cell line (Fig. 3B). We further determined whether the thyrospheres exhibit cisplatin chemoresistance by comparing the sensitivity of thyrospheres derived from THJ-11T and THJ-16T cells with that of the parental monolayer cells (Fig. 3C). After 24 hours in the presence of 10 µM of cisplatin, the survival rate of THJ-11T thyrospheres was ∼1.7-fold higher than that of THJ-11T monolayer cells (52.5 ± 3.5 *vs.* 30.0±1.4, *P*<0.05). In contrast, the survival rate of THJ-16T thyrospheres under identical conditions was similar to that of THJ-16T monolayer cells ((49.7±2.7 *vs.* 48.6±1.5, *P* = 0.73)(Fig. 3C). These results suggest that a subpopulation of spheroid-forming cells in THJ-11T cells, but not in THJ-16 cells, are resistant to cisplatin treatment.

### ATC cells initiate tumors in serially transplanted immunodeficient mice

To test our hypothesis that only a small population of CSCs is responsible for tumor formation, we transplanted each of the four ATC cell lines into groups of NOD/SCID *Il2rg-/-* mice — a highly immunocompromised strain that lacks T cells, B cells, and NK cells [12]. Table 1 shows that subcutaneous injection of any of the cell lines caused tumor formation in the mice. However, the length of time from injection to tumor formation, or the latency of the cell lines, varied: 28 days for THJ-11T, 28 to 70 days for THJ-16T, 56 to 77 days for THJ-21T, and 56 to 91 days for THJ-29T. These observations suggest that THJ-11T cells initiate tumor growth more efficiently than the other ATC cell lines.

**Table 1:** Limiting dilution analysis using human ATC cell lines in a subcutaneous mouse model of thyroid carcinoma.

Cell line	In vivo passage	Number cells injected	Incidence	Latency (days)
THJ-11T	primary	500,000	5/5	28
THJ-11T	secondary	500,000	5/5	27–28
THJ-11T	secondary	10,000	5/5	35
THJ-11T	secondary	1,000	5/5	28–35
THJ-11T	tertiary	500,000	5/5	7–14
THJ-11T	tertiary	10,000	5/5	21–28
THJ-11T	tertiary	1,000	4/5	35–42
THJ-16T	primary	500,000	4/5	28–70
THJ-16T	secondary	500,000	5/5	14
THJ-16T	secondary	10,000	5/5	28–49
THJ-16T	secondary	1,000	4/5	38–49
THJ-16T	tertiary	500,000	5/5	7
THJ-16T	tertiary	10,000	5/5	21–28
THJ-16T	tertiary	1,000	5/5	35–42
THJ-21T	primary	500,000	4/4	56–77
THJ-21T	secondary	500,000	5/5	14
THJ-21T	tertiary	500,000	5/5	7–14
THJ-21T	tertiary	10,000	5/5	35–36
THJ-21T	tertiary	1,000	3/4	49
THJ-29T	primary	500,000	3/3	56–91
THJ-29T	secondary	500,000	5/5	14–21
THJ-29T	secondary	10,000	4/5	28
THJ-29T	secondary	1,000	5/5	36–77
THJ-29T	tertiary	500,000	3/3	7
THJ-29T	tertiary	10,000	3/3	21
THJ-29T	tertiary	1,000	4/4	21
THJ-11T	thyrosphere	10,000	5/5	42–77
THJ-11T	thyrosphere	5,000	5/5	63–77
THJ-11T	thyrosphere	1,000	5/5	70–105

**Figure 4:**
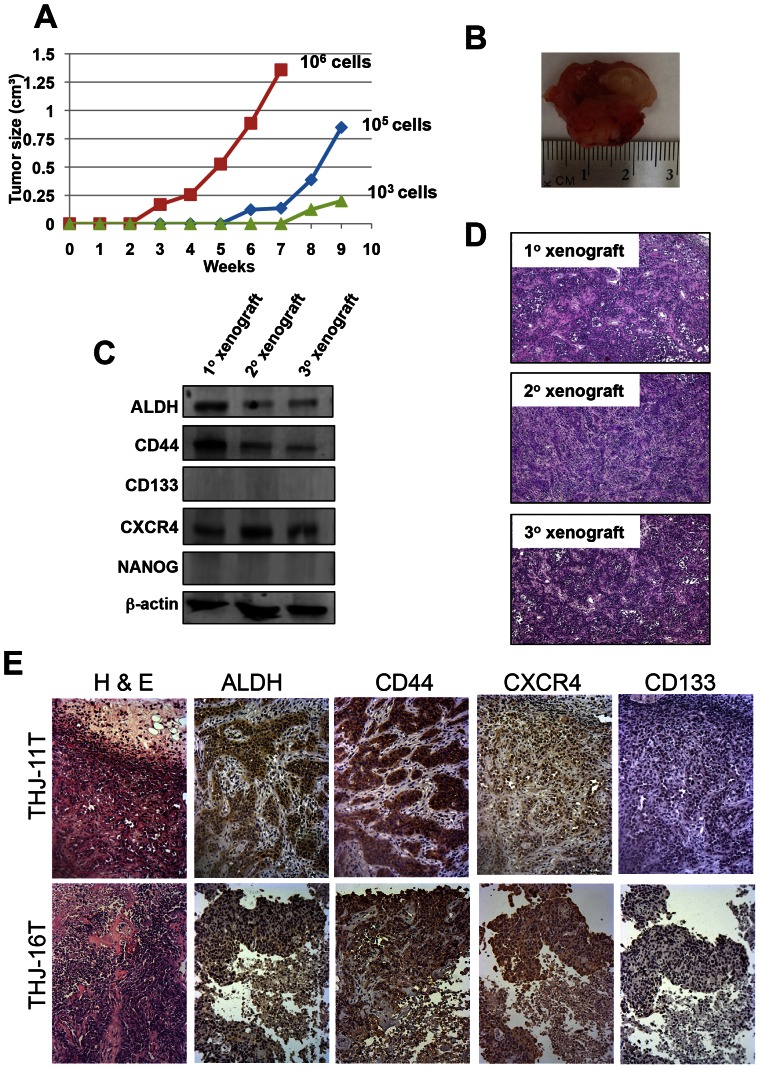
ATC monolayer-derived cells initiate tumors in serially transplanted immunodeficient mice. (A) Tumor growth curve generated by subcutaneous injection of THJ-11T parental monolayer-derived cells. The number of cells injected is indicated. (B) A representative subcutaneous tumor removed from a mouse xenograft. (C) Western blot analysis showing the expression of ALDH, CD44, and CXCR4 in cells derived from primary, secondary and tertiary xenografts. (D) H&E staining showed that tumors originating from primary, secondary and tertiary xenografts are composed of a highly heterogeneous population of cells with a large nucleus-to-cytoplasm ratio. (E) Immunohistochemical analysis of ALDH, CD44, CXCR4 and CD133 levels in mouse xenografts generated from primary xenografts of THJ-11T and THJ-16T. Note that mouse xenografts do not express CD133.

We next serially transplanted these primary xenografts into other NOD/SCID *Il2rg-/-* mice to determine the long-term tumorigenic potential of these cells. Limiting dilution transplantation experiments indicated that the rate of tumor growth increased with serial xenografting. For example, the latency of secondary xenografts of 5×10^5^ ATC cells (regardless of cell line) was 14 to 28 days, whereas that of an equivalent number of cells in tertiary xenografts was 7 to 14 days (Table 1). We also observed that the size of the subcutaneous tumors reflects the number of cells injected (Fig3. 4 A and B). Tumors derived from secondary and tertiary xenografts of THJ-11T cells consistently reproduced the primary tumors at the histological level, and Western blotting and immunohistochemical analysis confirmed the expression of tumor markers ALDH, CD44 and CXCR4 in the xenografts (Fig. 4 C-E). Note that mouse xenografts do not express CD133, consistent with a previous finding in primary cultures generated from surgical samples of human ATC [5]. Together, these results suggest that all four of these ATC cell lines have long-term tumorigenic potential and that our xenograft models quantitatively and qualitatively recapitulate tumorigenesis *in vivo*. The finding that each cell line can be propagated for three passages demonstrates their self-renewal potential *in vivo*. Notably, 42 to 77 days were required for tumor formation after subcutaneous injection of 10,000 THJ-11T thyrosphere-derived cells (Table 1). This increase in latency suggests that the subcutaneous space may not provide the appropriate microenvironment for thyrosphere-derived cells.

### ATC thyrosphere-derived cells are tumorigenic and metastasize to the thyroid and other surrounding tissues more aggressively than parental monolayer-derived cells in an orthotopic mouse model of thyroid carcinoma

**Figure 5:**
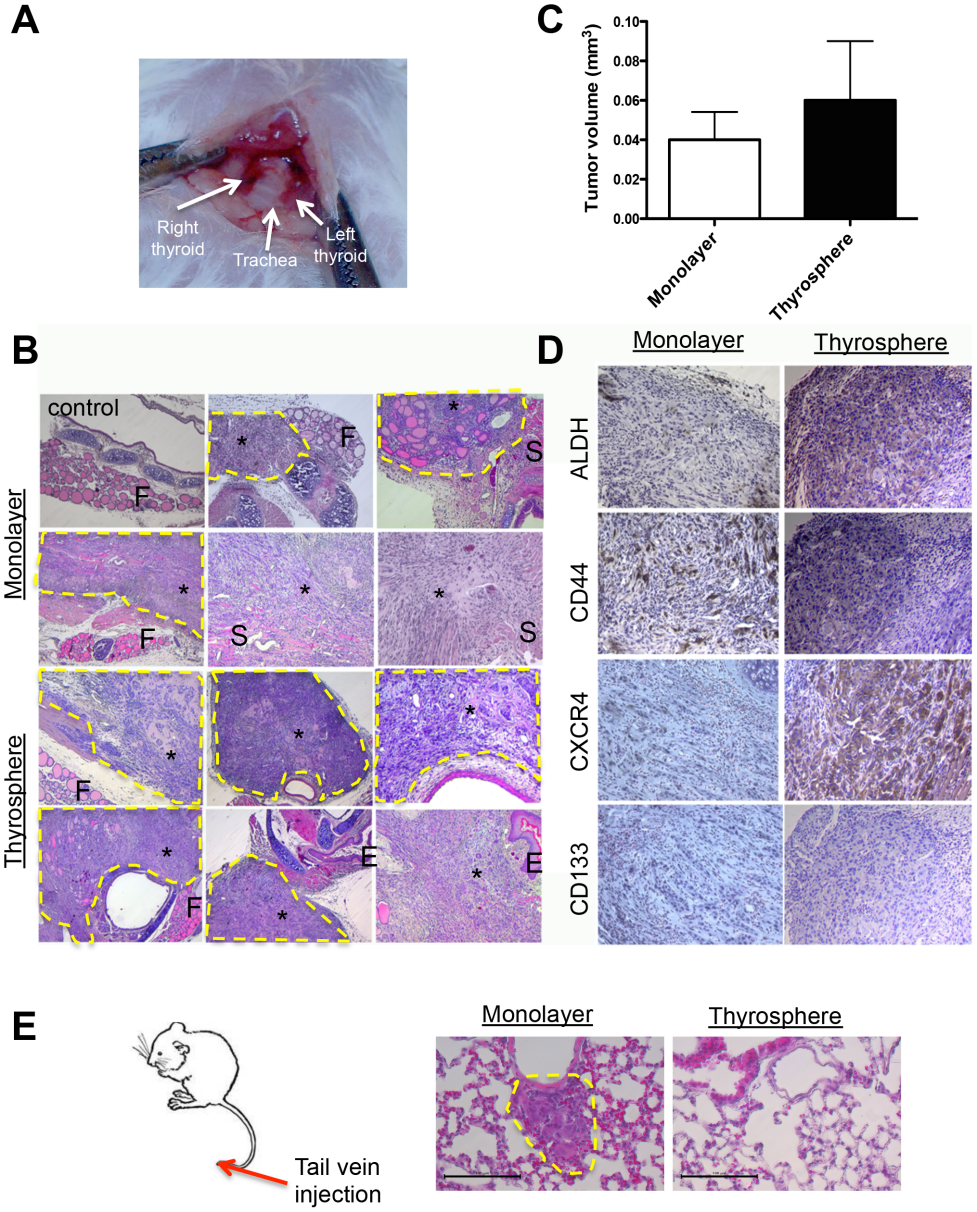
THJ-11T thyrosphere-derived cells are tumorigenic and metastasize more aggressively than parental monolayer-derived cells in an orthotopic mouse model of thyroid carcinoma. (A) One representative experiment showing the location of the thyroid gland and trachea. (B) Orthotopic tumors generated with 500,000 parental monolayer-derived cells and 10,000 thyrosphere-derived cells. H&E staining revealed the invasion of anaplastic tumors into the thyroid gland, trachea, smooth muscle and esophagus. F, normal thyroid follicles; S, smooth muscle; asterisk, thyroid tumors; E, esophagus. (C) Orthotopic tumors arising from thyrosphere-derived cells had a larger tumor volume than did those arising from parental monolayer-derived cells (60 ± 30 mm^3^ *versus* 40 ± 20 mm^3^; *P* = 0.25). (D) Immunohistochemical staining of ALDH, CD44, CXCR4 and CD133 in orthotopic tumors generated from monolayer- and thyrosphere-derived cells. Note the intense immunoreactivity of ALDH, CD44 and CXCR4 in thyrosphere-derived tumors four weeks post-injection. (E) Schematic representation of lung colonization assay by tail-vein injection model. Representative histology images of mice with lung metastasis induced by THJ-11T monolayer cells. Note that mice injected with thyrosphere-derived cells did not develop lung metastasis. Scale bar, 100 µm.

To further study ATC metastasis in a biological setting that closely mimics the disease process in humans, we established an orthotopic mouse model of thyroid carcinoma by directly injecting ATC cells into the thyroid gland (Fig. 5A). The orthotopic tumors established by this method invaded the trachea and muscle near the esophagus within two weeks of injection. After four weeks, the tumors displayed many clinical features of ATC — a high-grade malignant neoplasm characterized by a high mitotic index, nuclear atypia, cellular pleomorphism and necrosis. Five of five mice injected in the thyroid with 10,000 thyrosphere-derived cells developed tumors. Five of five mice injected with 500,000 THJ-11T cells (a 50-fold increase in cell number) cultured in monolayer also developed tumors. However, the thyrosphere-derived tumors grew faster and were larger four weeks after injection than were the tumors arising from the orthotopic injection of the vastly greater numbers of parental monolayer-derived cells (Fig. 5). The volumes of the thyrosphere-derived tumors were also substantially greater than those of monolayer-derived tumors (60±30 mm^3^ *vs.* 40±20 mm^3^; *P* = 0.25) (Figure 5C). Histological analysis of the orthotopic tumors arising from both thyrosphere and monolayer cells revealed extrathyroidal extension around the trachea, and tracheal invasion into smooth muscle and esophagus (Fig. 5B). Although immunohistochemical analysis confirmed the expression of ALDH, CD44 and CXCR4 in tumors derived from each cell type (Figure 5D) — a result consistent with the findings from the subcutaneous model (Fig. 4E) — a significantly greater percentage of cells in thyrosphere-derived tumors expressed these markers (Fig. 5D). Together, these observations validate the local metastatic potential of thyrosphere-derived cells to the thyroid and other neighboring tissues.

### ATC thyrosphere-derived cells do not induce lung metastasis

As the orthotopic transplantation model cannot always produce distant metastasis into the lung (a major cause of death in ATC), we injected 10,000 thyrosphere-derived cells or 500,000 THJ-11T cells cultured in monolayer into the tail veins of NOD/SCID *Il2rg-/-* mice to experimentally induce lung metastasis. Mice were sacrificed and lung sections were analyzed after six weeks. Numerous metastatic nodules were observed in the lungs of mice injected with THJ-11T cells cultured in monolayer, but none were found in the lungs of mice injected with thyrosphere-derived cells (Fig. 5E). These observations suggest that thyrosphere-derived cells do not promote the development of lung metastases in this experimentally induced lung metastasis model.

## Discussion

ATC is the most aggressive subtype of thyroid cancer. Due to its resistance to all types of cancer therapy, the median survival of ATC is only six months. However, the development of more-effective therapies has been hampered by a lack of validated thyroid cancer cell lines for large-scale drug screening. After a report in 2008 of serious thyroid cancer cell line cross-contamination issues [10], it became necessary to generate new, validated cell lines with a detailed characterization of cell-line integrity and STR profiles that genetically link the lines to their tissue of origin. The four ATC cell lines used in this study represent the first panel of thyroid cancer cell lines that meets these stringent new standards. Our results showed that all four ATC cell lines contain a small population of CSCs with self-renewal potential. We have further developed a simple and robust way to generate metastatic ATC in an orthotopic mouse model using human thyrospheres derived from these cell lines. Injection of the thyrosphere-derived cells into the thyroids of NOD/SCID *Il2rg-/-* mice resulted in the formation of metastatic tumors that recapitulated the clinical features of human ATC.

Thyroid CSCs are rare, and the absence of specific cell-surface markers makes their isolation challenging. Several markers, including CD44, ALDH and CD133, have been used successfully to isolate CSCs from breast, prostate, colorectal, glioblastoma, and pancreatic cancers [13]–[17], and CD133 has previously been identified as a putative CSC marker for ATC. However, publications reporting these observations have been criticized for their use of a contaminated colon cancer cell line [8], [9]. In the present study, none of the ATC cell lines expressed CD133, consistent with a previous finding in primary cultures generated from surgical samples of human ATC [5].

The advantages of ATC cell lines include the ability to culture them over long periods of time and to grow them in large quantities for high-throughput drug-screening applications. In spite of these advantages, it is necessary to confirm our findings in primary ATC cells because cell lines do not always recapitulate all aspects of primary tumors. However, the rarity and rapidly fatal nature of this malignancy has made this difficult to achieve in laboratory settings.

CSCs may be isolated through a variety of techniques, including flow cytometry based on the expression of specific cell-surface markers like those discussed above [18]–[21]. The sorting of side populations of cancer cells through Hoechst 33342 dye exclusion is an alternative approach [22]. Recent studies have also shown that the spheroid-forming assay and culture is an equally efficient method of separating CSCs from many solid tumors or cancer cell lines [23], particularly when — as is the case with ATC — a reliable cell-surface CSC marker has not yet been identified.

The thyrosphere assay is a well-studied *in vitro* stem cell assay to determine the clonality and multipotency of potential thyroid stem cells [2]. Dissociating thyrospheres into single cells, plating them to limiting dilution and then subjecting them to serial passaging in culture can further evaluate the long-term proliferation potential of these cells. Our data show that all four ATC cell lines can be cultured as thyrospheres and re-passaged multiple times, confirming their self-renewal potential *in vitro*. Our cytotoxicity studies showed that a small population of thyrospheres from THJ-11T cell line was resistant to cisplatin treatment. However, under the same conditions, thyrospheres and monolayer cells deriving from THJ-16T cells were equally sensitive to cisplatin. These differences could be due to the molecular and genetic differences between the two ATC cell lines and may warrant further investigation.

CSCs are able to reproduce the full heterogeneity of the parent tumor and grow continuously even after multiple passages. Thus, a definitive way to confirm the self-renewal and multipotency potential of a CSC *in vivo* is to regenerate the tumor in immunodeficient animals. We found that tumors from each of the four ATC cell lines not only recapitulated the histology and structure of the parent tumors, but could also be propagated *in vivo* for three passages. These results support our main hypothesis that some ATC cells exhibit stem cell properties *in vivo*. We further found that tertiary xenografts from all four ATC cell lines grew faster in NOD/SCID *Il2rg-/-* mice than did primary xenografts. Although these results may not necessarily indicate stem cell enrichment, they could indicate that some cancer cells in the tertiary xenografts are highly proliferative. Further investigation is needed to explain the more-aggressive tertiary tumor xenografts in ATC and to determine the implications, if any, for recurrent tumors in human patients.

In this study, we used NOD/SCID *Il2rg-/-* mice to test the ATC cell lines for xenograft growth. This mouse strain lacks mature T and B cells and is deficient for several high-affinity cytokine receptors — including IL2, IL4, IL7, IL9, IL15 and IL21 — required for the development of NK cells and innate immunity responses. As a result, the immune system of this strain is more severely impaired than that of the standard NOD/SCID mice. For example, it has been reported that one in four randomly selected single cells from a human melanoma sample could form a tumor in this mouse model, which is several orders of magnitude greater than the one-in-a-million cells suggested by studies in standard NOD/SCID mice [12]. Clearly, the selection of genetically modified mouse models for xenograft studies of putative CSCs can significantly affect the sensitivity of the assay and must be carefully considered when interpreting data. This NOD/SCID *Il2rg-/-* mouse strain has become the gold standard for testing CSC model due to its superior xenografting capability.

We have utilized three different transplantation approaches to study the tumorigenic and metastatic potential of thyrosphere-derived cells. Of the three transplantation sites we demonstrated in this report — subcutaneous, thyroid and tail vein — only the orthotopic thyroid transplantation model generated aggressive and metastatic tumors within two weeks of injection. In comparison, the subcutaneous model, although able to support tumor initiation, requires more than 40 days to generate detectable tumors from the same number of cells. Finally, the tail-vein injection model cannot initiate tumors after even six weeks. These observations suggest the existence of a niche in the thyroid that participates directly in the regulation of thyrosphere-derived cells. ATC metastasis is a complex and highly regulated process mediated by various tumor-derived factors. Understanding how thyrosphere-derived cells promote local, but not lung, metastasis may be crucial for developing more effective therapies for metastatic ATC.

In summary, the availability of ATC-specific thyrosphere-derived cells and the orthotopic tumor models that recapitulate the metastatic cancer so deadly to human patients gives thyroid cancer researchers a panel of unprecedented clinical tools. Our findings may prove useful in the elucidation of the molecular mechanisms underlying the dissemination of metastatic CSCs and the exploration of therapeutic strategies that directly target thyroid CSCs.

## Materials and Methods

### Human ATC cell culture, colony formation assay, and thyrosphere assay

The human ATC cell lines THJ-11T, THJ-16T, THJ-21T and THJ-29T [11] were provided by Dr. John A. Copland (Mayo Clinic). Cells were cultured in RPMI-1640 medium (Cellgro, Manassas, VA) supplemented with 10% fetal bovine serum (FBS), nonessential amino acids, sodium pyruvate, and penicillin-streptomycin-amphotericin B. Cultures were maintained in a humidified chamber in a 5% CO_2_/air mixture at 37°C. For the colony-formation assay, cells were cultured in the methylcellulose-based media MethoCult according to the manufacturer's instructions (Stemcell Technologies, Vancouver, Canada). For the spheroid-forming assay, single cells were plated at 5,000 cells/well on ultra-low-attachment six-well plates (Fisher Scientific Co., Hampton, NH). Spheres were counted after seven days. The percentage of cells that form thyrospheres is calculated from the total number of cells seeded. The numerator is the number of thyrospheres formed per well, and the denominator is 5,000.

For *in vitro* serial passaging, thyrospheres were collected by gentle centrifugation at 800 rpm for 5 min and dissociated enzymatically with 0.05% trypsin/EDTA. The dissociated cells were passed through 40 µm mesh filters (BD Falcon Cell Strainer, Franklin Lakes, NJ) to eliminate doublets or triplets. Single cells were plated at 5,000 cells/well on ultra-low-attachment six-well plates to generate secondary thyrospheres. Three such rounds of serial passage were performed. In some experiments, spheres were collected after seven days, trypsinized into single-cell suspensions and then mixed with Matrigel/RPMI in a 1∶1 dilution for injection into mice.

### RNA isolation and qRT-PCR

Total RNA was isolated from 1×10^6^ cells with the RNeasy kit (Qiagen, Valencia, CA) and treated with RNase-free DNase (Qiagen). Two micrograms of total RNA were reverse transcribed into cDNA using the Thermoscript First Strand Synthesis System (Invitrogen, Grand Island, NY). The oligonucleotide sequences of the primers (*Pax8*, *TTF1*, *TSHR*, *TG*, *NIS* and *TPO*) have been published elsewhere [24], [25]. The mRNA levels were quantified in triplicate by qRT-PCR on a ViiA7 PCR System (Applied Biosystems, Foster City, CA). PCR was performed with a SYBR Green PCR Master mix. Human GAPDH was used as the housekeeping gene during the amplifications.

### *In vivo* tumorigenicity experiments

Eight-week-old female NOD/SCID *Il2rg^-/-^* mice were obtained from Taconic Farms Inc. and maintained under specific pathogen-free conditions with the approval of the Institutional Animal Care and Use Committee of Saint Louis University School of Medicine. For subcutaneous transplantation, single cells were re-suspended in 100 µl of Matrigel/RPMI in 1∶1 dilution and injected subcutaneously into NOD/SCID *Il2rg^-/-^* mice. Tumor burden was measured twice a week by palpation and calipers, and tumor volumes were calculated by the following formula: (π/6)×large diameter ×(small diameter)^2^. The mice were monitored for three to five months for the appearance and development of tumors. Mice were sacrificed when the tumors reached 1.5 cm^3^.

For serial passage, tumors were harvested, minced, collagenase-digested and passed through 40 µm mesh filters to obtain a single-cell suspension. The resulting cell population was termed “secondary passage” and was cultured for three to seven days in RPMI/10% FBS medium and then re-inoculated into NOD/SCID *Il2rg^-/-^* mice. Subsequent tumors were used for repeated rounds of ATC cell isolation and the generation of additional serially-passaged ATC cells up to three passages. Separate fragments of tumors were removed, fixed in 4% paraformaldehyde and embedded in paraffin in preparation for immunohistochemistry.

For orthotopic transplantation, mice were anesthetized using ketamine and xylazine. The neck of each mouse was shaved and the skin and subcutaneous tissues were incised with scissors. 10 µl of cells was injected into the right thyroid gland. After the injection, the incision was closed using nylon sutures. Antibiotic ointment was applied to the wound and the mice were placed under a warming lamp while they recovered from anesthesia. Mice were sacrificed two and four weeks after injection. Tumors and adjacent tissues were collected and analyzed for metastasis by histology and immunohistochemistry.

For tail-vein injection, lung metastases were induced by injection of 500,000 monolayer cells or 10,000 thyrosphere cells into the lateral tail vein. After six weeks, mice were sacrificed and lung sections were analyzed by H&E staining.

### Alamar blue assay for cell proliferation

ATC cells were seeded in triplicate into 96-well plates at a concentration of 10^4^ cells/well in RPMI/10% FBS and placed in a humidified chamber of 5% CO_2_/air mixture at 37°C. For cell proliferation assays, Alamar Blue dye (Invitrogen) was added directly to the culture media to a final concentration of 10%. After one hour of incubation, the plates were read in a fluorescent plate reader. As a negative control, Alamar blue was added to cell-free medium.

### Western blot analysis

Cells were washed twice with PBS and resuspended in 0.5 ml lysis buffer. An equal amount of protein from each sample was resolved by SDS/PAGE followed by immunobotting with anti-ALDH1A2 (1∶500; Sigma), CD44 (1∶1000; Sigma), CD133/1 (1∶100; Miltenyi Biotec), CXCR4 (1∶1000; Sigma), NANOG (1∶500; Sigma) and β-actin (1∶2000; Sigma) antibodies. Anti-IgG conjugated with horseradish peroxidase was used as a secondary antibody. The membranes were developed using a chemiluminescence system (ECL detection reagent; Amersham Pharmacia).

### Immunohistochemistry and immunofluorescence

Immunohistochemistry was performed on paraformaldehyde-fixed, paraffin-embedded tissue specimens. Paraffin sections were de-paraffined and rehydrated with distilled water. The slides were subsequently incubated with anti-human ALDH, CD44, CXCR4, CD133 antibodies (Abcam) and visualized with Elite Vector Stain ABC systems (Vector Laboratories) and DAB substrate (DakoCytomation) and counterstained with hematoxylin. For indirect immunofluorescence, cells were fixed in 4% paraformaldehyde in PBS. After fixation, the cells were washed and permeabilized in PBS containing 0.1% Triton X-100 for 10 minutes, then pre-blocked with 3% BSA for one hour. The following primary antibodies were used: rabbit anti-human Nanog (1∶100) and rabbit anti-human Oct4 (1∶100). For detection of primary antibodies, the cells were washed, and then incubated with a Texas Red- or FITC-conjugated goat polyclonal to rabbit IgG antibody (1∶100) for 30 minutes at room temperature. The stained cells were washed before mounting with 10 µl Vectashield mounting medium containing 4′, 6-diamidino-2-phenylindole (DAPI; Vector Laboratories). Images were captured using a Leica DMI3000B inverted fluorescent microscope using Leica AF6000E software (North Central Instruments). Confocal microscopy images were captured with Olympus FV-1000 MPE multiphoton laser scanning microscope on an upright platform.

### Statistical analysis

Statistical analysis was performed with GraphPad Prism 5.0 software. Numerical data are expressed as mean ± s.e.m. Statistical differences are considered significant at *P*<0.05.

## References

[1] Ain KB (1999) Anaplastic thyroid carcinoma: a therapeutic challenge. Semin Surg Oncol 16: 64–69. 9890741 10.1002/(sici)1098-2388(199901/02)16:1<64::aid-ssu10>3.0.co;2-u

[2] Lin RY (2011) Thyroid cancer stem cells. Nat Rev Endocrinol 7: 609–616. 21788969 10.1038/nrendo.2011.127

[3] Mitsutake N, Iwao A, Nagai K, Namba H, Ohtsuru A, et al (2007) Characterization of side population in thyroid cancer cell lines: cancer stem-like cells are enriched partly but not exclusively. Endocrinology 148: 1797–1803. 17234707 10.1210/en.2006-1553

[4] Thomas D, Friedman S, Lin RY (2008) Thyroid stem cells: lessons from normal development and thyroid cancer. Endocr Relat Cancer 15: 51–58. 18310275 10.1677/ERC-07-0210 PMC2673699

[5] Todaro M, Iovino F, Eterno V, Cammareri P, Gambara G, et al (2010) Tumorigenic and metastatic activity of human thyroid cancer stem cells. Cancer Res 70: 8874–8885. 20959469 10.1158/0008-5472.CAN-10-1994

[6] Zhang P, Zuo H, Ozaki T, Nakagomi N, Kakudo K (2006) Cancer stem cell hypothesis in thyroid cancer. Pathol Int 56: 485–489. 16930327 10.1111/j.1440-1827.2006.01995.x

[7] Zheng X, Cui D, Xu S, Brabant G, Derwahl M (2010) Doxorubicin fails to eradicate cancer stem cells derived from anaplastic thyroid carcinoma cells: characterization of resistant cells. Int J Oncol 37: 307–315. 20596658 10.3892/ijo_00000679

[8] Zito G, Richiusa P, Bommarito A, Carissimi E, Russo L, et al (2008) In vitro identification and characterization of CD133(pos) cancer stem-like cells in anaplastic thyroid carcinoma cell lines. PLoS ONE 3: e3544. 18958156 10.1371/journal.pone.0003544 PMC2568821

[9] Friedman S, Lu M, Schultz A, Thomas D, Lin RY (2009) CD133+ anaplastic thyroid cancer cells initiate tumors in immunodeficient mice and are regulated by thyrotropin. PLoS ONE 4: e5395. 19404394 10.1371/journal.pone.0005395 PMC2671400

[10] Schweppe RE, Klopper JP, Korch C, Pugazhenthi U, Benezra M, et al (2008) Deoxyribonucleic acid profiling analysis of 40 human thyroid cancer cell lines reveals cross-contamination resulting in cell line redundancy and misidentification. J Clin Endocrinol Metab 93: 4331–4341. 18713817 10.1210/jc.2008-1102 PMC2582569

[11] Marlow LA, D′Innocenzi J, Zhang Y, Rohl SD, Cooper SJ, et al (2010) Detailed molecular fingerprinting of four new anaplastic thyroid carcinoma cell lines and their use for verification of RhoB as a molecular therapeutic target. J Clin Endocrinol Metab 95: 5338–5347. 20810568 10.1210/jc.2010-1421 PMC2999968

[12] Quintana E, Shackleton M, Sabel MS, Fullen DR, Johnson TM, et al (2008) Efficient tumour formation by single human melanoma cells. Nature 456: 593–598. 19052619 10.1038/nature07567 PMC2597380

[13] Dalerba P, Dylla SJ, Park IK, Liu R, Wang X, et al (2007) Phenotypic characterization of human colorectal cancer stem cells. Proc Natl Acad Sci U S A 104: 10158–10163. 17548814 10.1073/pnas.0703478104 PMC1891215

[14] Collins AT, Berry PA, Hyde C, Stower MJ, Maitland NJ (2005) Prospective identification of tumorigenic prostate cancer stem cells. Cancer Res 65: 10946–10951. 16322242 10.1158/0008-5472.CAN-05-2018

[15] Al-Hajj M, Wicha MS, Benito-Hernandez A, Morrison SJ, Clarke MF (2003) Prospective identification of tumorigenic breast cancer cells. Proc Natl Acad Sci U S A 100: 3983–3988. 12629218 10.1073/pnas.0530291100 PMC153034

[16] Kang MK, Kang SK (2007) Tumorigenesis of chemotherapeutic drug-resistant cancer stem-like cells in brain glioma. Stem Cells Dev 16: 837–847. 17999604 10.1089/scd.2007.0006

[17] O′Brien CA, Pollett A, Gallinger S, Dick JE (2007) A human colon cancer cell capable of initiating tumour growth in immunodeficient mice. Nature 445: 106–110. 17122772 10.1038/nature05372

[18] Ginestier C, Hur MH, Charafe-Jauffret E, Monville F, Dutcher J, et al (2007) ALDH1 is a marker of normal and malignant human mammary stem cells and a predictor of poor clinical outcome. Cell Stem Cell 1: 555–567. 18371393 10.1016/j.stem.2007.08.014 PMC2423808

[19] Yu C, Yao Z, Dai J, Zhang H, Escara-Wilke J, et al (2011) ALDH activity indicates increased tumorigenic cells, but not cancer stem cells, in prostate cancer cell lines. In Vivo 25: 69–76. 21282737

[20] Beier D, Hau P, Proescholdt M, Lohmeier A, Wischhusen J, et al (2007) CD133(+) and CD133(−) glioblastoma-derived cancer stem cells show differential growth characteristics and molecular profiles. Cancer Res 67: 4010–4015. 17483311 10.1158/0008-5472.CAN-06-4180

[21] Wright MH, Calcagno AM, Salcido CD, Carlson MD, Ambudkar SV, et al (2008) Brca1 breast tumors contain distinct CD44+/CD24− and CD133+ cells with cancer stem cell characteristics. Breast Cancer Res 10: R10. 18241344 10.1186/bcr1855 PMC2374965

[22] Ho MM, Ng AV, Lam S, Hung JY (2007) Side population in human lung cancer cell lines and tumors is enriched with stem-like cancer cells. Cancer Res 67: 4827–4833. 17510412 10.1158/0008-5472.CAN-06-3557

[23] Pastrana E, Silva-Vargas V, Doetsch F (2011) Eyes wide open: a critical review of sphere-formation as an assay for stem cells. Cell Stem Cell 8: 486–498. 21549325 10.1016/j.stem.2011.04.007 PMC3633588

[24] Presta I, Arturi F, Ferretti E, Mattei T, Scarpelli D, et al (2005) Recovery of NIS expression in thyroid cancer cells by overexpression of Pax8 gene. BMC Cancer 5: 80. 16029487 10.1186/1471-2407-5-80 PMC1180821

[25] Boldarine VT, Maciel RM, Guimaraes GS, Nakabashi CC, Camacho CP, et al (2010) Development of a sensitive and specific quantitative reverse transcription-polymerase chain reaction assay for blood thyroglobulin messenger ribonucleic acid in the follow-up of patients with differentiated thyroid carcinoma. J Clin Endocrinol Metab 95: 1726–1733. 20173019 10.1210/jc.2009-1354

